# Development of novel monoclonal antibodies for blocking NF-κB activation induced by CD2v protein in African swine fever virus

**DOI:** 10.3389/fimmu.2024.1352404

**Published:** 2024-05-23

**Authors:** Rongrong Fan, Zeliang Wei, Mengmeng Zhang, Shanshan Jia, Zhiyang Jiang, Yao Wang, Junyu Cai, Guojiang Chen, He Xiao, Yinxiang Wei, Yanchun Shi, Jiannan Feng, Beifen Shen, Yuanqiang Zheng, Yaojiang Huang, Jing Wang

**Affiliations:** ^1^ Laboratory for Genetic Engineering of Antibodies and Functional Proteins, Beijing Institute of Pharmacology and Toxicology, Beijing, China; ^2^ Beijing Engineering Research Center of Food Environment and Public Health, Minzu University of China, Beijing, China; ^3^ Inner Mongolia Key Laboratory of Molecular Biology, Inner Mongolia Medical University, Hohhot, China; ^4^ Joint National Laboratory for Antibody Drug Engineering, The First Affiliated Hospital of Henan University, Henan University, Kaifeng, China; ^5^ BCA Bio-Breeding Center, Beijing Capital Agribusiness Co., Ltd., Beijing, China

**Keywords:** ASFV-CD2v, eukaryotic expression, monoclonal antibodies, glycosylation, NF-κB, epitope

## Abstract

**Background:**

CD2v, a critical outer envelope glycoprotein of the African swine fever virus (ASFV), plays a central role in the hemadsorption phenomenon during ASFV infection and is recognized as an essential immunoprotective protein. Monoclonal antibodies (mAbs) targeting CD2v have demonstrated promise in both diagnosing and combating African swine fever (ASF). The objective of this study was to develop specific monoclonal antibodies against CD2v.

**Methods:**

In this investigation, Recombinant CD2v was expressed in eukaryotic cells, and murine mAbs were generated through meticulous screening and hybridoma cloning. Various techniques, including indirect enzyme-linked immunosorbent assay (ELISA), western blotting, immunofluorescence assay (IFA), and bio-layer interferometry (BLI), were employed to characterize the mAbs. Epitope mapping was conducted using truncation mutants and epitope peptide mapping.

**Results:**

An optimal antibody pair for a highly sensitive sandwich ELISA was identified, and the antigenic structures recognized by the mAbs were elucidated. Two linear epitopes highly conserved in ASFV genotype II strains, particularly in Chinese endemic strains, were identified, along with a unique glycosylated epitope. Three mAbs, 2B25, 3G25, and 8G1, effectively blocked CD2v-induced NF-κB activation.

**Conclusions:**

This study provides valuable insights into the antigenic structure of ASFV CD2v. The mAbs obtained in this study hold great potential for use in the development of ASF diagnostic strategies, and the identified epitopes may contribute to vaccine development against ASFV.

## 1 Introduction

Both wild and domestic pigs are susceptible to African swine fever (ASF), a highly contagious and fatal disease caused by the African swine fever virus (ASFV) (1, 2). ASFV causes acute hemorrhagic fever in these hosts, with a mortality rate of up to 100% (3). Despite extensive research efforts, there is no effective vaccine against the disease. ASFV was first reported in Kenya in the 1920s (4), and genotype II then emerged in Georgia in 2007, spreading to the Russian Federation and Eastern Europe (5–7). In 2018, ASFV spread to China, world’s largest producer of pigs, and subsequently to other Asian countries, resulting in economic losses of approximately USD 111.2 billion in 2019 (8, 9). ASF has become widespread globally, inflicting significant economic losses on the global swine industry (10).

ASFV belongs to the ASFV genus, which consists of a single species. Its genome ranges between 170 and 193 kilobase pairs (11–14) and encodes 68 structural proteins and over 100 non-structural proteins (15, 16). The virions have an icosahedral shape with a multilayer structure, including an internal core, internal lipid membrane, icosahedral capsid, and outer lipid envelope (10). In harsh environments and under protein-rich conditions, it is sufficient for long-term survival (17). However, the limited understanding of the functions of most of the ASFV proteins has hindered vaccine development. Due to the absence of available vaccines or treatments, the current control measures primarily involve strict quarantine and biosecurity practices, which heavily restrict animal movement and require the slaughter of affected animals.

**Figure 1:**
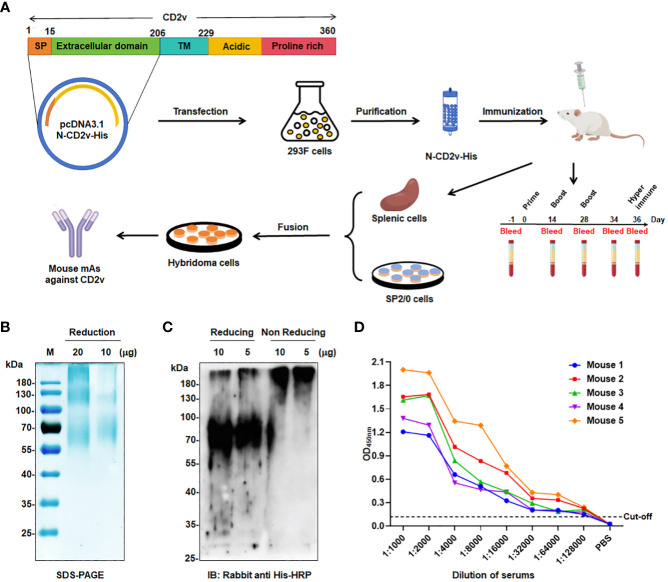
Generation of mAbs against ASFV-CD2v protein. **(A)** Schematic representation of the generation process for anti-CD2v mAbs. **(B)** SDS-PAGE analysis of purified recombinant N-CD2v-His protein in a 12% gel. **(C)** Western blotting of N-CD2v-His protein using anti-6×His tag antibody, under reducing or non-reducing conditions. **(D)** Indirect ELISA of the serum titers of five mice immunized with N-CD2v-His, with the serum of unimmunized mice serving as the negative control. The cut-off values were calculated based on the mean+3 SD of the optical density (OD) value of the negative control.

*EP402R* encodes the CD2v protein, which is similar to the CD2 protein in the host (18). CD2v is composed of a signal peptide, a transmembrane region, two immunoglobulin-like domains, and a variable number of proline-rich repeats in the cytoplasmic domain (**Figure 1A**). As a glycoprotein (gp110–140), CD2v is inserted into the outer lipid envelope and it is the only viral protein that can be detected on the surface of extracellular virions. During the late stage of infection, CD2v is highly expressed and its extracellular domain acts as a key mediator in the hemadsorption process, thereby facilitating viral transport and evasion of the immune system (19–21). CD2v interacts with two adaptor proteins, clathrin adaptor protein 1 (AP-1) (22) and actin-binding adaptor protein (DBNL, also known as SH3P7) (23). Through these interactions, cellular transport mechanisms can be modified, contributing to Golgi reorganization. Additionally, CD2v hinders the translocation of STING from the endoplasmic reticulum to the Golgi body, thereby preventing STING activation (24). CD2v establishes interactions with surrounding lymphocytes and macrophages via lymphocyte function-associated antigen-3 (LFA-3/CD58), promoting the activation of nuclear factor (NF)-κB. This activation subsequently upregulates interferon (IFN)-β and IFN-stimulated genes (ISGs), such as 2’5’-oligoadenylate synthetase (OAS) proteins, while also potentially activating other intrinsic or extrinsic apoptotic pathways, ultimately leading to the apoptosis of lymphocytes and macrophages (25).

CD2v is an immunoprotective antigen that has been identified as crucial in the context of ASFV. It plays a significant role in inducing serotype-specific cross-protective immunity and contributes to the mediation of serological specificity through hemagglutination inhibition (21, 26, 27). Research using various virus models has provided valuable insights into the role of CD2v in virulence and immunoprotection (28, 29). Notably, some ASFV strains possess truncated or interrupted *EP402R* genes, resulting in the loss of their hemadsorption ability, and they often exhibit an attenuated phenotypic profile and have been utilized as a promising model for vaccine research (30). Despite the induction of specific antibodies and cellular immunity by viral vaccines, achieving optimal immunoprotection in pigs remains an unresolved challenge (31–33).

In recent years, the development of monoclonal antibodies (mAbs) targeting linear epitopes of CD2v has made progress, though their bioactivity has not been evaluated extensively (**Table 1**) (34–38). Hence, we opted for the Expi293F eukaryotic expression system to express the recombinant N-CD2v-His protein (extracellular region of CD2v), as it offers superior post-translational modifications, particularly glycosylation, ensuring that the antigens expressed closely resemble the *in vivo* structure of CD2v (**Figure 1A**). As a result, we successfully generated three specific mAbs with a high affinity, as indicated in **Table 2**. These mAbs displayed strong reactivity towards glycosylated and/or deglycosylated CD2v. Additionally, we established a highly sensitive sandwich ELISA configuration. Furthermore, our study identified two conserved linear B-cell epitopes, along with a unique glycosylated epitope. Most importantly, our findings demonstrated that the identified mAbs effectively blocked CD2v-induced NF-κB activation.

**Table 1:** Identification of B-cell epitopes on ASFV-CD2v protein.

ASFV strain	Antigen structure	Expression system	B-cell epitopes	Experiment	Ref.
China/2018/Anhui XCGQ strain (GenBank: MK128995.1)	CD2v dimeric protein with His tag	Baculovirus expression system in Sf21 cells	^147^FVKYT^151^	I-ELISA, Dot-ELISA	(34)
			^157^EYNWN^161^		
			^195^SSNY^198^		
ASFV-SY18 strain (GenBank: MH766894)	CD2v fused with Norovirus P particle assembled into nanoparticle	HEK293F cells	^28^LDSNITNDNNDINGVSWNFFNNSF^51^	IFA	(35)
China/2018/Anhui XCGQ strain (GenBank: MK128995.1)	CD2v extracellular domain with C-terminal His tag	CHO-S cells	^128^TCKKNNGTNT^137^	I-ELISA, Dot-ELISA	(36)
			^148^VKYTNESILE^157^		
China/2018/Anhui XCGQ strain (GenBank: MK128995.1)	CD2v extracellular domain with C-terminal 6×His tag	Eukaryotic expression system	^38^DINGVSWN^45^	I-ELISA, Dot-ELISA	(37)
			^134^GTNTNIY^140^		
ASFV-SY18 strain (GenBank accession MH766894)	CD2v extracellular domain	HEK293F cells	^154^SILEYNW^160^	Dot-blot; Peptide-based ELISA	(38)
			^154^SILE^157^		
ASFV Georgia 2007/1 strain (GenBank: NC044959)	NA	NA	^160^WNNSNINNFT^169^	Bioinformatics prediction, Dot-ELISA	(39)
ASFV Pig/HLJ/2018 strain (GenBank ID MK333180.1)	CD2v intracellular region with N-terminal 6×His tag	Prokaryotic expression system	^264^EPSPREP^270^	ELISA, Dot-blot	(40)

NA, not applicable.

**Table 2:** Summary of anti-CD2v mAbs generated in this study.

mAb	Western blotting		IFA	ELISA	Epitope region	K_D_ (nM)	NF-κB blocking activity
	Glyco	deGlyco					
2B25	+	−	+	+	AA16–48^*^	15.4	+
3J25	+	+	+	+	^25^TIILDSNITNDNN^37^	9.94	+
8G1	+	+	+	+	^141^LNINDTFVKYTNE^153^	23.5	+

Glyco, glycosylation; deGlyco, deglycosylation; AA, amino acid; +, positive; −, negative.

^*^, glycosylated epitope.

Taken together, our results demonstrate that mAbs have significant potential as valuable tools for conducting ASFV CD2v structure–function studies, and other applications. Moreover, our results provide a foundation for the development of novel ASFV diagnostic reagents and therapeutic strategies.

## 2 Materials and methods

### 2.1 Cell lines, plasmids, and animals

Human embryonic kidney 293T (HEK293T) cells, porcine kidney cells (PK15), and Sp2/0 myeloma cells were obtained from the American Type Culture Collection (ATCC, USA). The HEK293T cells were incubated in Dulbecco’s modified eagle medium (DMEM), supplemented with 10% fetal bovine serum (FBS) (Gibco, USA). The HEK293T and PK15 cells were maintained at 37°C with 5% CO_2_. The eukaryotic expression plasmid pcDNA3.1-CD2v-HA containing a hemagglutinin (HA)-tagged full-length coding sequence of the *EP402R* gene (encoding CD2v) from the ASFV SY-1 strain (GenBank: OM161110.1; Chinese strain; genotype II ASFV) was obtained from GenScript Biotech Co, Ltd. (Nanjing, China). The pEGFP-N1-CD2v-GFP plasmid containing a green fluorescent protein (GFP)-tagged full-length coding sequence of the *EP402R* gene was constructed by our laboratory based on pcDNA3.1-CD2v-HA. From Beijing Vital River Laboratory Animal Technology Co., Ltd (Beijing, China), we purchased female specific-pathogen-free BALB/c mice. The animal experiments were conducted in accordance with national guidelines and were approved by the Ethics Committee of the Academy of Beijing Institute of Pharmacology and Toxicology (IACUC-DWZX-2021–621).

### 2.2 Main reagents

We purchased restriction endonucleases *Xba*I and *Not*I from New England Biolabs Inc. (USA). Roswell Park Memorial Institute (RPMI) 1640 medium, DMEM, MEM, hypoxanthine–aminopterin–thymidine (HAT) selective medium, An Expi293 expression system kit (including Expi293F cells, Expi293 expression medium, ExpiFectamine 293 transfection kit, and Opti-MEM I antiserum medium), Alexa 594-labeled Goat anti-mouse IgG, and mouse IgG (mIgG) as the isotype control, etc. were purchased from Thermo Fisher Scientific (USA). Freund’s complete adjuvant, Freund’s incomplete adjuvant, tunicamycin, and Triton X-100 were purchased from Sigma-Aldrich (USA). Enhanced chemiluminescence (ECL) solution was purchased from Cytiva Co., Ltd. (USA). Radioimmunoprecipitation assay (RIPA) cell lysis buffer was purchased from Solarbio technology Co., Ltd, (Beijing, China). Human and mouse CD2 with His tag (hCD2-His and mCD2-His) were purchased from ACROBiosystems (USA). Recombinant ASFV-P30, P72 and pp62 protein with His tag were sourced from East-Mab Bio Technology (Suzhou, China). Rabbit anti-6×HA tag antibody was purchased from R&D Systems (USA). Rabbit anti-His tag antibody was purchased from Abcam (USA). Horseradish peroxidase (HRP)-labeled goat anti-mouse and anti-rabbit IgG secondary antibodies and 4′,6­diamidino­2′­phenylindole (DAPI) were purchased from ZSGB Biotechnology Co., Ltd. (Beijing, China). Rabbit antibodies against phosphorylated NF-κB p65 (p-NF-κB p65), NF-κB p65, glyceraldehyde 3-phosphate dehydrogenase (GAPDH), and β-actin were purchased from Cell Signaling Technology Inc. (USA).

### 2.3 Construction of eukaryotic expression plasmid expressing the CD2v extracellular domain

To construct the eukaryotic expression plasmid expressing the CD2v extracellular domain (amino acids 16–206), we amplified the target fragment using the pcDNA3.1-CD2v-HA plasmid as a template. To enhance the expression and secretion efficiency, we replaced the original signal peptide (amino acids 1–15) with a signal peptide sequence derived from IL-10 (MHSSALLCCLVLLTGVRA). Additionally, to enable convenient downstream purification, we introduced a C-terminal 6×His tag. After digestion with the *Not*I and *Xba*I restriction enzymes, the gene fragment corresponding to the CD2v extracellular domain was directly cloned into the pcDNA3.1(+) eukaryotic expression plasmid, which was designated pcDNA3.1-N-CD2v-His. The recombinant expression plasmid was verified by double enzyme digestion and sequencing.

### 2.4 Expression and purification of the CD2v extracellular domain fusion protein

Expression of the CD2v extracellular domain fusion protein (N-CD2v-His) was conducted following the guidelines provided in the Expi293 expression system kit (Thermo Fisher Scientific, USA). Briefly, a 50-mL cell culture was prepared in a 125-mL cell shake flask. Expi293F cells were added to the flask and diluted to a concentration of 3 × 10^6^ cells/mL. The expression plasmid (pcDNA3.1-N-CD2v-His) and ExpiFectamine 293 transfection reagents were diluted with Opti-MEM. The transfection reagents were mixed with the diluted plasmid and added to the flask. After incubation for 18–22 h, ExpiFectamine 293 transfection enhancers were added. The cells were then cultured for an additional 5–7 days. The culture supernatant, which contained N-CD2v-His, was filtered using a 0.45-μm filter membrane. Purification of the protein was carried out using a Ni-NTA His Bind protein purification kit (GenScript Biotech Co, Ltd., Nanjing, China). Following elution, the protein was concentrated by ultrafiltration, and its concentration was determined using a bicinchoninic acid (BCA) protein concentration determination kit (Thermo Fisher Scientific, USA). Finally, the purified protein was analyzed using sodium dodecyl sulfate-polyacrylamide gel electrophoresis (SDS-PAGE), followed by detection using western blotting with a rabbit anti-His tag antibody.

### 2.5 Preparation of and screening for anti-CD2v mAbs

Anti-CD2v mAbs were generated following a previously described method (41). Anti-CD2v mAbs were generated as described (15). Female BALB/c mice were subcutaneously immunized with 100 µg purified N-CD2v-His protein per mouse in combination with Freund’s adjuvant, followed by two biweekly booster immunizations using the same dose and incomplete adjuvant. Antibody titer in serum were measured by ELISA. Mice received a final hyperimmunization dose with 100 µg/mouse intraperitoneally, and were euthanized 3 days later. Splenocytes were fused with Sp2/0 myeloma cells using PEG 1500. Positive hybridomas were subcloned and high-producing lines were injected into mice to induce ascites. Purification was achieved using a HiTrap Protein G HP column (GE Healthcare, Cytiva, USA).

### 2.6 Identification of anti-CD2v mAb subclasses and isotypes

To perform mAb subclass analysis, a mouse antibody isotype detection ELISA kit (Thermo Fisher Scientific, USA) was used following the manufacturer’s instructions. Hybridoma cell total RNA was isolated using an RNeasy Plus Micro Kit (QIAGEN, Germany) as instructed by the manufacturer. The isolated RNA was then reverse transcribed into cDNA using SMARTScribe Reverse Transcriptase (TaKaRa, Japan) according to the manufacturer’s instructions. Heavy and light chain antibody fragments were amplified using a previously reported protocol (42) and sent to GenScript Biotech Co., Ltd. (Nanjing, China) for sequencing. The complementarity-determining region 3 (CDR3) sequences were verified using the IMGT online software (https://imgt.org/IMGT_vquest/input).

### 2.7 Detection of binding titers of anti-CD2v mAbs by indirect ELISA

ELISA plates were coated with 0.2 μg/well of N-CD2v-His and incubated overnight at 4°C. The plates were then blocked with 4% skim milk in PBST (PBS with 0.1% Tween-20) at 37°C for 1 hour. Gradient dilutions of purified mAbs and a negative control mouse IgG isotype were added to the wells and incubated at 37°C for 2 hours. After washing with PBST, HRP-labeled goat anti-mouse IgG secondary antibody (1:3000 dilution) was added and incubated at 37°C for 45 minutes. Tetramethylbenzidine (TMB) solution (Kangwei Century Biotechnology Co., Ltd., Beijing, China) was then added for visualization. The optical density (OD) at 450 nm was measured using an ELISA microplate reader.

### 2.8 Detection of mAb affinity by bio-layer interferometry

To determine the affinity of anti-CD2v mAbs, we utilized a ForteBio-Octet molecular interaction analyzer with the BLI technique. First, N-CD2v-His was immobilized onto Ni-NTA biosensors. Then, the mAbs were diluted and added to the biosensors to bind to N-CD2v-His. The binding and dissociation times were 200 and 400 s respectively. To regenerate the biosensor surfaces, a pH 1.7 solution of 10 mM Gly-HCl was applied in 5-s pulses, repeated three times. The data obtained were analyzed using a 1:1 binding model to calculate the equilibrium dissociation constant (K_D_).

### 2.9 Detection of mAb specificity by western blotting

HEK293T cells were transfected with pcDNA3.1-CD2v-HA or empty plasmid pcDNA3.1 (control), using jetPRIME transfection reagent (Polyplus, France) for 12 h. The cells were then incubated with or without 10 μg/ml tunicamycin for 24 h (22, 43). To extract the cellular proteins, RIPA buffer containing a protease inhibitor cocktail (R&D, USA) was used for cell lysis. The protein lysates were separated by SDS-PAGE on 12% gels and transferred onto polyvinylidene fluoride (PVDF) membranes. Subsequently, the membranes were submerged in blocking buffer (5% skim milk in PBST) for 1h at 37°C, incubated with mAbs or a rabbit anti-HA tag primary antibody (positive control) at 4°C overnight, washed with PBST, and then incubated for 1 h at room temperature with HRP- labeled goat anti-rabbit IgG secondary antibody (diluted in blocking buffer). After washing with PBST again, the bound antibodies were detected using an ECL western blotting detection system.

### 2.10 Detection of CD2v and mAb localization by IFA

HEK293T cells were inoculated in 35mm confocal dishes (1 × 10^5^ cells/dish). When the cells grew to about 70% confluence, a pEGFP-N1-CD2v-GFP plasmid or an empty pEGFP-N1 plasmid (negative control) were transfected into the cells. After 36 h, 4% paraformaldehyde was added for fixation for 24 h at 4°C. Next, absorbed paraformaldehyde and dialysate (1% bovine serum albumin [BSA]+0.1% Triton X-100/PBS buffer) were added for permeabilization for 20 min at room temperature. Dialysate containing 5% goat serum was then added and incubated for 1 h at 37°C. Thereafter, 1 mg/mL of each of the selected mAbs was separately added and incubated overnight at 4°C. Alexa 594-labeled goat anti-mouse IgG secondary antibody was added and incubated at room temperature in the dark for 1 h. Next, 1 drop of DAPI was added and the fluorescence localization in cells was observed by confocal microscopy.

### 2.11 Competitive ELISA and sandwich ELISA

To evaluate the overlapping epitopes recognized by different anti-CD2v mAbs, competitive ELISA was conducted. The selected mAbs (2B25, 3J25, and 8G1) were biotinylated using an EZ-Link™ Sulfo-NHS-Biotinylation kit (ThermoFisher, USA). N-CD2v-His protein (0.2 µg/well) was coated onto 96-well ELISA plates overnight at 4°C. The plates were then blocked with 4% skim milk in PBST at 37°C for 1 hour. Varying concentrations (35.2 ng/mL to 36 μg/mL) of a competitor mAb (2B25, 3J25, or 8G1) and 1 μg/mL of a biotinylated detection mAb (2B25-biotin, 3J25-biotin, or 8G1-biotin) were added to each well and incubated at 37°C for 1 hour. After washing, streptavidin–HRP conjugate (1:4000 dilution, Thermo Fisher, USA) was added and incubated at 37°C for 45 minutes. TMB solution was added, and the reaction was stopped by adding H2SO4. The OD at 450 nm was measured as before.

To develop a sensitive sandwich ELISA for CD2v detection, the reactivity of the anti-CD2v mAbs as capture and detection antibodies was evaluated. Each mAb (1 μg/mL) was coated separately onto an ELISA plate. Serially diluted N-CD2v-His protein (1.9 ng/mL to 2 μg/mL) was added to the plate. After washing, each biotinylated mAb (1 μg/mL) was added to the appropriate wells. After washing again, streptavidin–HRP conjugate (1:8000 dilution in blocking buffer) was added and incubated at 37°C for 45 minutes. TMB solution was added, and the reaction was stopped using 1N H_2_SO_4_. The OD at 450 nm was measured.

### 2.12 Identification of ASFV CD2v B-cell epitopes

Based on the sequence characteristics of the CD2v extracellular region, obtained from the Swiss Model database (https://swissmodel.expasy.org), we utilized secondary structure discrimination and potential epitope prediction analysis to design, express, and purify five truncation mutants (CD2v D1–D5) of the CD2v extracellular domain. To identify the CD2v regions recognized by mAbs, we initially conducted an indirect ELISA. Based on these results, we designed 11 overlapping peptides that spanned the CD2v extracellular domain (specifically, D1 and D4). To facilitate better coupling, cysteine residues were added at the C-terminus of the shorter peptides. These peptides were synthesized and conjugated to Bovine Serum Albumin (BSA) by Chinese Peptide (Hangzhou, China), ensuring their purity was confirmed to be ≥95%. To further investigate the binding sites, a peptide-based ELISA was performed. The 96-well plates were coated with the peptides (0.1 μg/well) at 4°C overnight and then blocked with 4% skim milk at 37°C for 1 h. Each mAb (0.1 μg/well) was added separately and incubated for 37°C for 1 h. Next, an HRP-labeled goat anti-mouse IgG secondary antibody (1:3000 dilution) was added and incubated at 37°C for 45 min. Subsequently, the plates were processed following the same steps as described above for indirect ELISA. To construct the theoretical 3D structure of the CD2v extracellular domain, we used the model with the highest sequence similarity (PDB code: 2dru). The optimized model was visualized using PyMol software.

### 2.13 Conserved analysis of epitopes among different ASFV strains

To evaluate the conserved nature of epitope sequences among different ASFV strains, 15 Chinese epidemic strains and 27 representative globally circulating strains (comprising 5 genotypes) were selected, with strain information presented in **Supplementary Table S1**. The conservation of mAb-binding CD2v epitope amino acid sequences in different ASFV strains was assessed by multiple sequence alignment in Jalview v2.11.3.2 (44). A maximum likelihood phylogenetic tree was constructed based on the CD2v amino acid sequence of the 42 representative ASFV strains in MEGA v11.0 (45) and visualized utilizing ggtree v3.10.0 (46).

### 2.14 Impact of mAbs on the CD2v-induced activation of the NF-κB signaling pathway

In a time course experiment, PK-15 cells were incubated with 500 ng of N-CD2v-His and PBS (negative control) for 15–120 minutes to study CD2v-induced NF-κB activation. Cell lysis was performed using RIPA buffer containing a protease and phosphatase inhibitor cocktail (R&D, USA), followed by western blotting using specific rabbit primary antibodies to detect phosphorylated NF-κB p65 (p-NF-κB p65), total NF-κB p65, and the reference protein GAPDH. To assess the inhibitory activity of mAbs on CD2V-induced NF-κB signaling activation, different concentrations of each mAb (2–16 μg) were preincubated with 500 ng of N-CD2v-His protein for 30 min at 37°C. The mAb and CD2v mixture were then added to PK-15 cells and incubated for 90 min at 37°C. After cell lysis, western blotting was performed to detect phosphorylated NF-κB p65, total NF-κB p65, and the reference protein β-actin using specific rabbit primary antibodies.

### 2.15 Statistical analyses

Statistical analyses were conducted using Prism v8.0 (GraphPad, San Diego, CA, USA). The experimental data are presented as mean ± standard deviation (SD). Unpaired *t*-tests were used for statistical analyses, and differences were considered significant at p < 0.05.

## 3 Results

### 3.1 Preparation of CD2v protein and generation of four anti-CD2v mAbs

Anti-CD2v mAbs were generated using the hybridoma technique through a series of selection and cloning processes (**Figure 1A**; **Supplementary Figure S1**). Initially, the recombinant CD2v extracellular domain was purified, and then SDS-PAGE and western blotting confirmed that it was highly glycosylated, with an approximate molecular weight of 55–80 kDa, with the main band at around 70 kDa (**Figures 1B, C**). This is consistent with previous findings (22, 23, 43). Subsequently, five BALB/c mice were immunized with the purified protein, and serum samples were collected for antibody titer detection using indirect ELISA. The serum from all five mice exhibited high titers against the N-CD2v-His glycosylated protein, indicating strong immunogenicity (**Figure 1D**). Hybridoma cell lines were generated by fusing mouse splenocytes from the three mice with the highest titers with Sp2/0 cells. Initially, a total 158 hybridoma cell lines that showed affinity to CD2v were obtained. Following two rounds of subcloning, sequencing analysis, and the exclusion of clones with repetitive sequences, we carefully selected four hybridoma cell lines (2B25, 3J25, 7B1, and 8G1) based on their superior binding affinity for further investigation. The purified antibodies were identified using SDS-PAGE (**Supplementary Figure S2**). The isotypes of mAbs were determined to be IgG2b for 2B25 and IgG1 for the other three mAbs. Moreover, all antibodies had kappa light chains (**Table 3**; **Supplementary Figure S3**). The V gene subclasses and the CDR3 sequence for each mAb are presented in **Table 3**.

**Table 3:** Identification of anti-CD2v mAb subclasses.

mAb	Chain	Isotype	V gene	CDR3	Germline V gene identity
2B25	H	IgG2b	IGHV3–1	ARLGGVDY	97.92%
	L	κ	IGKV4–68	QQWTSNPFT	95.29%
3J25	H	IgG1	IGHV5–6-5	VRGYRYYSMDY	91.93%
	L	κ	IGKV4–57-1	QRYSGNPPIT	96.81%
7B1	H	IgG1	IGHV1–74	ATAFFDY	91.32%
	L	κ	IGKV1–135	WQGTHFPQT	98.64%
8G1	H	IgG1	IGHV5–9-1	ARSNPYYFDY	98.26%
	L	κ	IGKV1–135	WQGTHFPT	97.96%

### 3.2 Preliminary analysis of binding activity of anti-CD2v mAbs and specificity to CD2v using indirect ELISA

**Figure 2:**
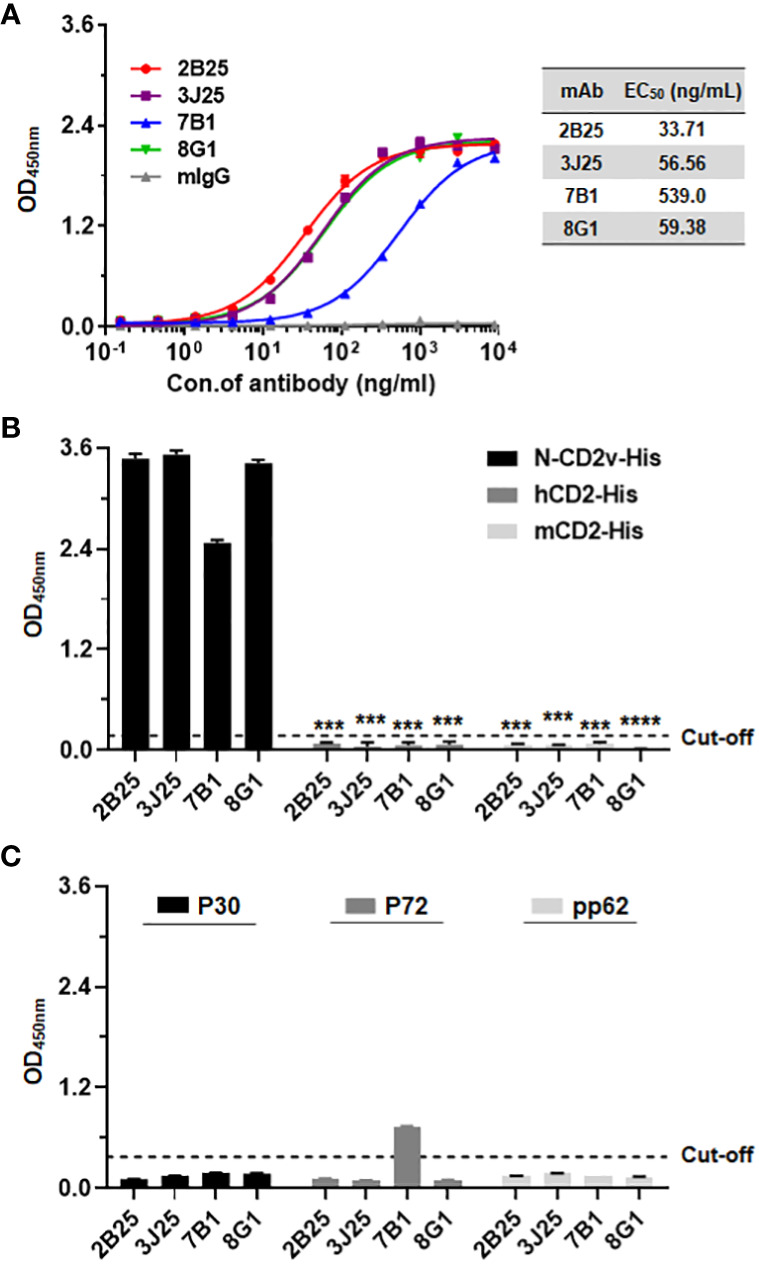
Analysis of antibody–antigen binding and specificity. **(A)** Determination of four mAbs (2B25, 3J25, 7B1, and 8G1) binding titer to N-CD2v-His by indirect ELISA. The results are presented as the mean ± SD of experiments performed in triplicate. **(B)** Preliminary analysis of mAbs specificity for N-CD2v-His compared to hCD2-His and mCD2-His using indirect ELISA. ***: p <0.001, ****: p <0.0001. **(C)** Analysis of the binding interactions of the four mAbs with ASFV structural proteins P30, P72, and pp62 through indirect ELISA.

Using indirect ELISA, the N-CD2v-His binding activity of the mAbs was confirmed. All four mAbs (2B25, 3J25, 8G1, and 7B1) showed dose-dependent binding, with half maximal effective concentration (EC_50_) values of 33.71, 56.32, 59.23, and 539.10 ng/mL, respectively. The mouse IgG isotype control antibody exhibited no binding (**Figure 2A**). To assess the specificity of the four mAbs for CD2v, known for its high similarity to CD2 found in T cells (18), their binding to hCD2 and mCD2 was evaluated using ELISA. The results clearly revealed that all four mAbs specifically bound to N-CD2v-His but no binding to hCD2-His or mCD2-His (**Figure 2B**). Furthermore, we investigated the binding interactions of the four mAbs with ASFV structural proteins P30, P72, and pp62, respectively. The results revealed that 2B25, 3J25, and 8G1 did not exhibit significant binding to any of the three ASFV structural proteins. Conversely, antibody 7B1 showed no binding to P30 and pp62, but displayed partial cross-reactivity with P72 (**Figure 2C**). These findings indicate that the screened mAbs, 2B25, 3J25, and 8G1, are specifically targeted towards the CD2v antigen.

### 3.3 Analysis of anti-CD2v mAb affinity using BLI

**Figure 3:**
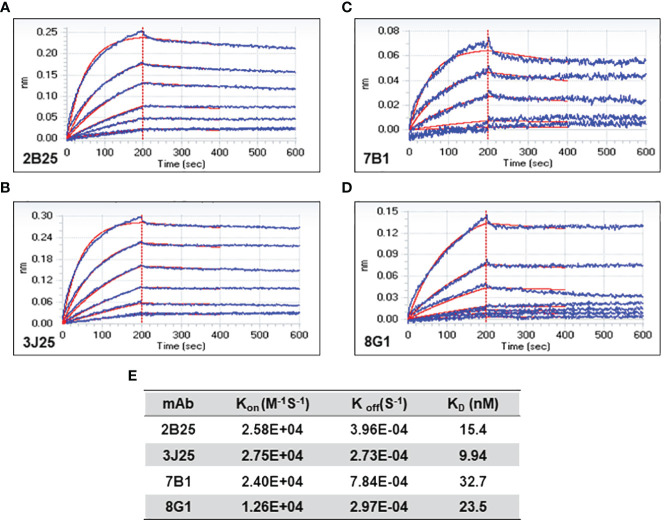
Bio-layer interferometry (BLI) analysis of anti-CD2v mAb affinity. **(A–D)** Association and dissociation curves for 2B25, 3J25, 7B1, and 8G1, respectively. **(E)** Summary of kinetic constants (K_on_, K_off,_ and K_D_) of the four mAbs.

The affinity of the four mAbs was evaluated using BLI (47, 48). The results demonstrated favorable association and dissociation curves for all four mAbs with the CD2v antigen at the nanomolar level (**Figure 3**). The results demonstrated that 3J25 exhibited the lowest K_D_ value (9.94 nM), indicating the highest affinity among the four mAbs, while 7B1 exhibited the highest K_D_ value (32.7 nM), indicating the lowest affinity. In the subsequent experiments, we primarily focused on the three mAbs with higher affinity to the CD2v antigen (2B25, 3J25, and 8G1).

### 3.4 Characterization of anti-CD2v mAbs using western blotting assay and IFA

**Figure 4:**
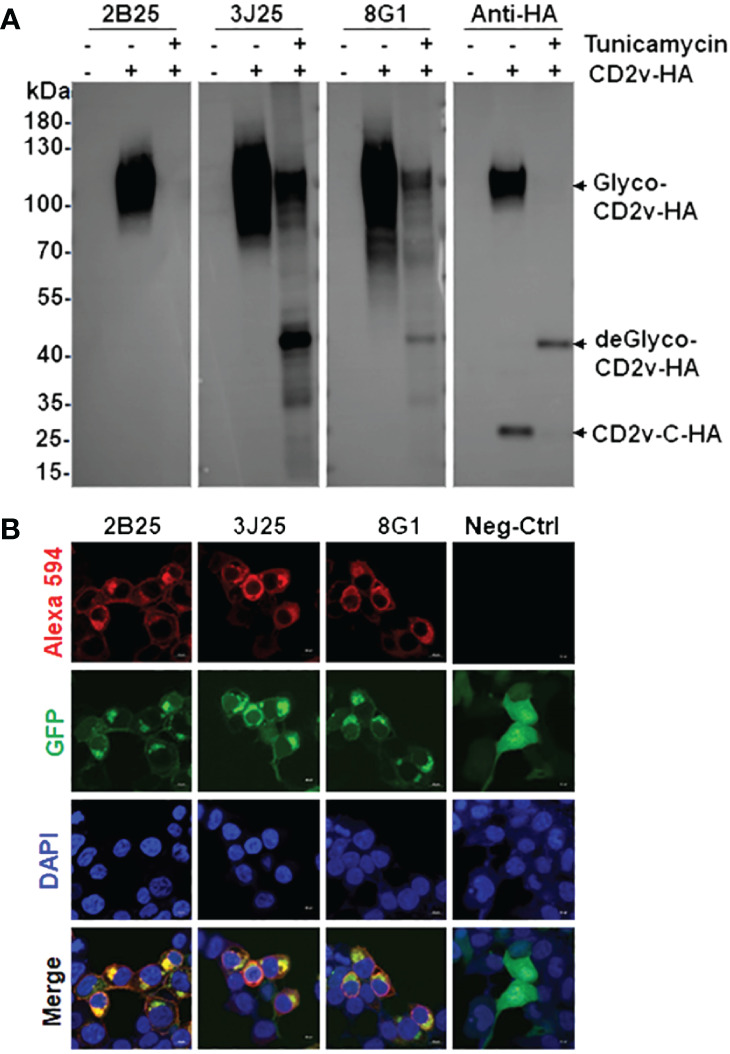
Characterization and identification of mAbs by western blotting and immunofluorescence assay (IFA). **(A)** Western blotting assay of the binding of the four mAbs to exogenous full-length CD2v-HA protein. After transfection with pcDNA3.1-CD2v-HA plasmid, HEK293T cells were treated with or without 10 μg/mL tunicamycin for 24 (h) Glyco: glycosylated, deGlyco-: deglycosylated, CD2v-C-HA: intracellular C-terminal region of CD2v. **(B)** IFA of the cellular localization of exogenous full-length CD2v-GFP protein and mAbs. The empty pEGFP-N1 plasmid served as the negative control (Neg-Ctrl). Scale bar, 10 µm.

In the western blotting assay of the binding of the three mAbs (2B25, 3J25, and 8G1) to exogenous CD2v-HA, HEK293T cells were transfected with a pcDNA3.1-CD2v-HA plasmid encoding the full-length CD2v protein fused with a C-terminal HA tag (**Figure 4A**). The results revealed that all three mAbs effectively recognized the glycosylated CD2v-HA full-length protein (about 100 kDa), but only 3J25 and 8G1 recognized the deglycosylated CD2v protein (about 43 kDa). Notably, none of the mAbs recognized the intracellular C-terminal region of CD2v (26 kDa) (**Figure 4A**). These findings highlight the dependence of the recognition of CD2v by 2B25 on the presence of the glycosylated epitope on the surface of CD2v. Furthermore, the findings suggest that the three of mAbs could target linear epitopes on CD2v.

In the IFA of the cellular localization of CD2v, we transfected HEK293T cells with a pEGFP-N1-CD2v-GFP plasmid expressing full-length CD2v with a C-terminal GFP tag. The results showed that CD2v-GFP exhibited green fluorescence and predominantly localized to the cytoplasmic and perinuclear regions, as well as the cytomembranes (**Figure 4B**). This localization pattern confirms previous findings (22, 23, 25, 43). Simultaneously, the mAbs, employed as primary antibodies, displayed red fluorescence upon detection using Alexa 594-labeled secondary antibody. Notably, there was significant colocalization between the red signal from the mAbs and the green signal from CD2v-GFP in the merged image (**Figure 4B**). These results provide evidence that all three mAbs specifically recognize the naturally spatially structured CD2v-GFP protein expressed in HEK293T cells.

### 3.5 Competitive ELISA assessing the epitope overlap of anti-CD2v mAbs and the development of sandwich ELISA

**Figure 5:**
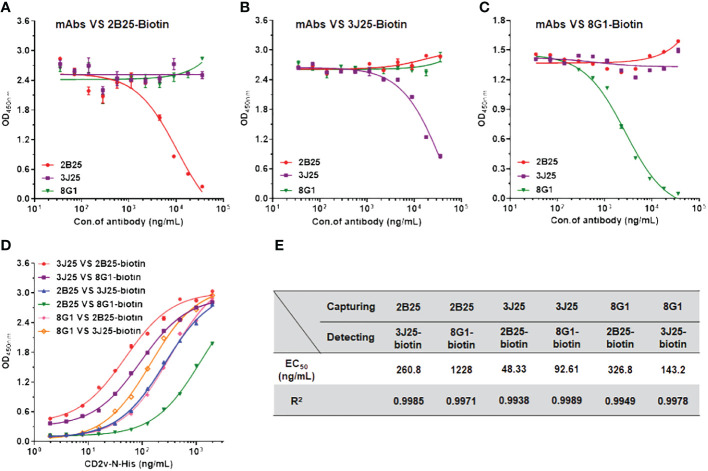
Identification of potential epitope overlaps using competitive ELISA and development of sandwich ELISA. **(A–C)** Competitive ELISA was performed to assess the potential overlap of epitopes recognized by different anti-CD2v mAbs. Varying concentrations (35.2 ng/mL to 36 μg/mL) of an unconjugated mAb (2B25, 3J25, or 8G1; competitor antibody) and 1 μg/mL of a biotinylated mAb (2B25, 3J25, or 8G1; detection antibody) were added to wells precoated with N-CD2v-His protein and allowed to compete for antigen binding. **(D)** Sandwich ELISA was conducted using various concentrations (1.9 ng/mL to 2 μg/mL) of N-CD2v-His as the antigen and various combinations of capture and detection antibodies. **(E)** Summary of EC_50_ values for the various combinations of capture and detection antibodies.

Competitive ELISA was employed to elucidate the relationships of the epitopes recognized by the three mAbs. N-CD2v-His was immobilized on ELISA plates, followed by adding an mAb (2B25, 3J25, or 8G1; competitor antibody) at varying concentrations and then a biotinylated mAb (2B25-biotin, 3J25-biotin, or 8G1-biotin; detection antibody), allowing for competition for binding to CD2v. As the concentration of 2B25 increased, the binding of 2B25-biotin to CD2v gradually decreased but minimal changes were observed regarding the binding of 3J25-biotin and 8G1-biotin (**Figure 5A**). This indicates that the epitopes recognized by 2B25 do not overlap with those recognized by 3J25 and 8G1. Similar results were obtained when using 3J25 or 8G1 as the competitor antibody (**Figures 5B, C**). Therefore, it can be concluded that 2B25, 3J25, and 8G1 do not exhibit epitope overlap. To establish a sandwich ELISA, an orthogonal experiment was conducted with these mAbs (**Figures 5D, E**). By utilizing 3J25 as the capture antibody and 2B25-biotin as the detection antibody, successful detection of viral ASFV-CD2v antigen at ng/mL levels was achieved.

### 3.6 Analysis of CD2v epitopes bound by anti-CD2v mAbs

**Figure 6:**
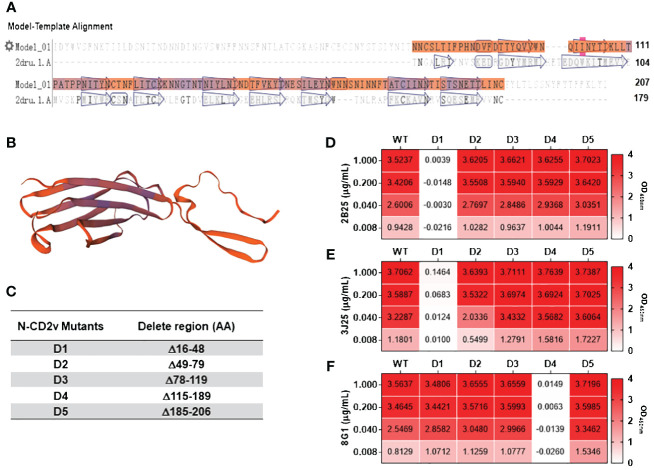
Preliminary analysis of the CD2v extracellular domain regions recognized by mAbs. **(A)** Sequence analysis of the CD2v extracellular domain was performed using the SwissModel server (https://swissmodel.expasy.org). **(B)** Homology modeling of the CD2v extracellular domain was conducted using PyMol software based on the model with the highest sequence similarity (PDB code: 2dru). **(C)** Design of five CD2v extracellular domain truncation mutants (D1–D5). **(D–F)** Indirect ELISA of mAbs 2B25, 3J25, and 8G1, respectively, recognizing D1–D5 using different concentrations of D1–D5.

Based on the sequence characteristics of the CD2v extracellular region (**Figures 6A, B**) and using secondary structure discrimination and potential epitope prediction analysis, we designed, expressed, and purified five CD2v extracellular domain truncation mutants (CD2v D1–D5; **Figure 6C**; **Supplementary Figure S4**). Indirect ELISA (**Figures 6D-F**) showed that 2B25 and 3J25 mainly recognized the D1 region. However, at high CD2v concentrations (1 μg/mL), there was a slight binding of 3J25 to the D1 region compare the negative control; at low CD2v concentrations (0.008 μg/ml), there was a slight lower recognition of the D1 region by 3J25 than 2B25. This indicates that 2B25 and 3J25 have subtle differences regarding their recognition of the fine epitopes within the N-terminal D1 region of CD2v. Additionally, 8G1 predominantly recognized the D4 region, which is different from the other two mAbs.

**Figure 7:**
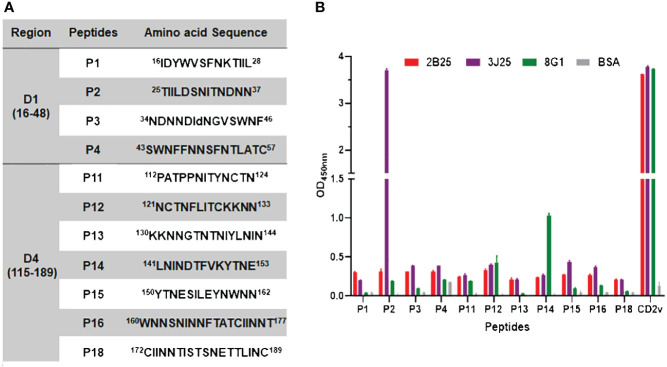
Epitope peptide mapping for mAbs recognizing CD2v. **(A)** Design of overlapping polypeptides with their corresponding amino acid sequences. **(B)** Indirect ELISA of three mAbs (2B25, 3J25, and 8G1) binding to peptides conjugated with BSA. ELISA plates were coated with 0.1 μg/well of the respective peptides, followed by adding each of the three mAbs separately. N-CD2v-His served as a positive control, while BSA served as a negative control. The experiment was performed in triplicate.

**Figure 8:**
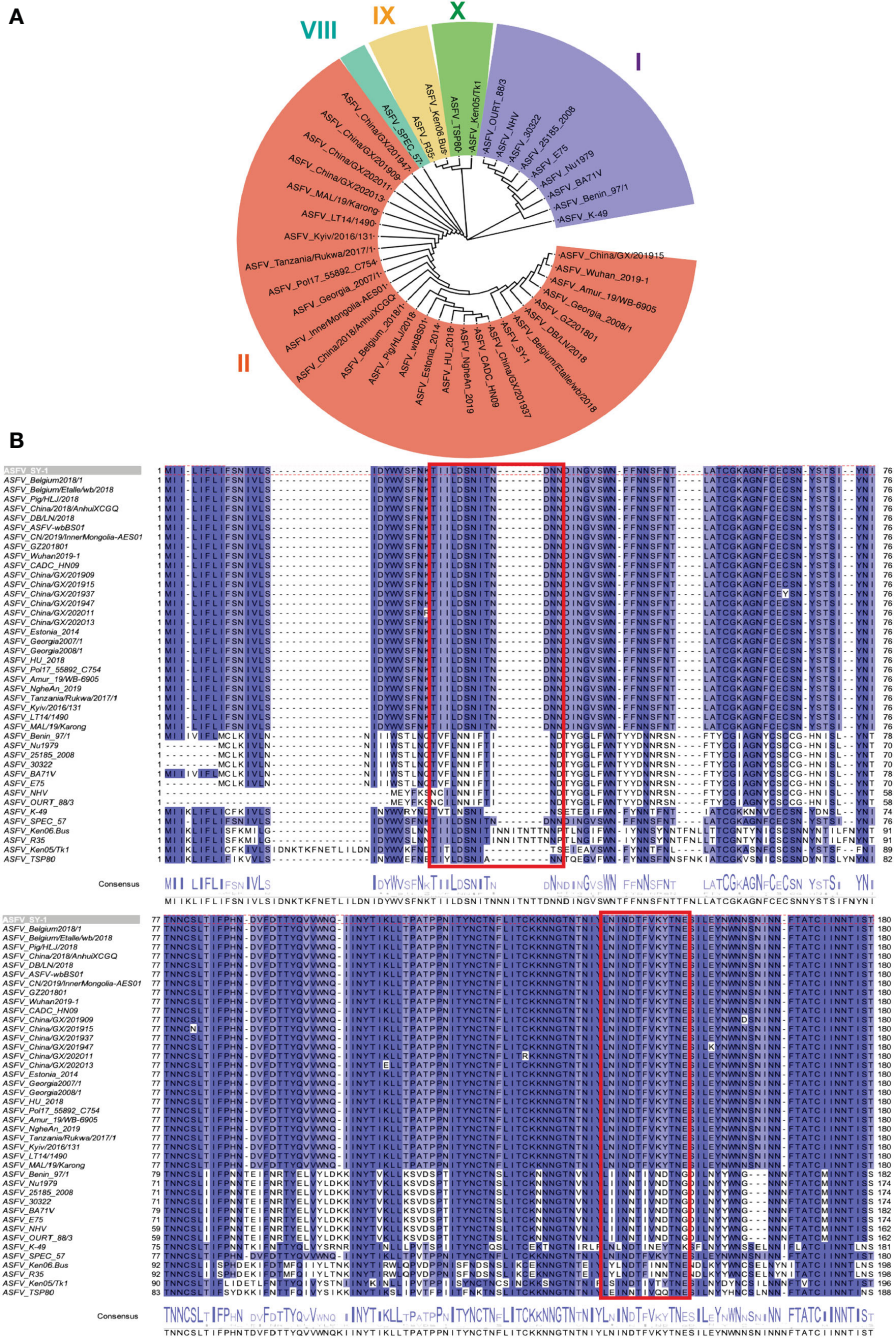
Conservation analysis of CD2v extracellular domains and epitopes across representative ASFV strains. **(A)** Maximum likelihood phylogenetic tree was built in MEGA v11.0 based on the CD2v protein sequences of the 42 representative strains. **(B)** Conservation of epitopes based on multiple sequence alignment of CD2v protein sequences in Jalview v2.11.1.4. The red box indicates the antigenic region, including ^25^TIILDSNITNDNN^37^ and ^141^LNINDTFVKYTNE^153^ epitopes.

To distinguish the epitopes recognized by these three mAbs, we designed 11 overlapping polypeptides spanning the D1 and D4 regions (**Figure 7**). The results revealed that 3J25 primarily recognized the P2 region, while 8G1 recognized the P14 region. Intriguingly, 2B25 did not exhibit binding affinity towards any of the peptides tested, indicating its potential recognition of glycosylated epitopes due to the absence of glycosylation modifications in the peptides. Thus, through epitope analysis, we have identified two novel linear B-cell epitopes (^25^TIILDSNITNDNN^37^ and ^141^LNINDTFVKYTNE^153^). Furthermore, when analyzing the conservation of these epitopes among 42 epidemic strains of ASFV (**Supplementary Table S1**), we observed high conservation within Chinese epidemic strains and Georgia 2008/1 strains, but differences compared to Benin97/1, Nu1979, E75, BA71V, K-49 strains, among others (**Figure 8**). Overall, our results underscore distinct epitope recognition patterns exhibited by the three mAbs.

### 3.7 Anti-CD2v mAbs inhibit CD2v-induced NF-κB signaling activation

**Figure 9:**
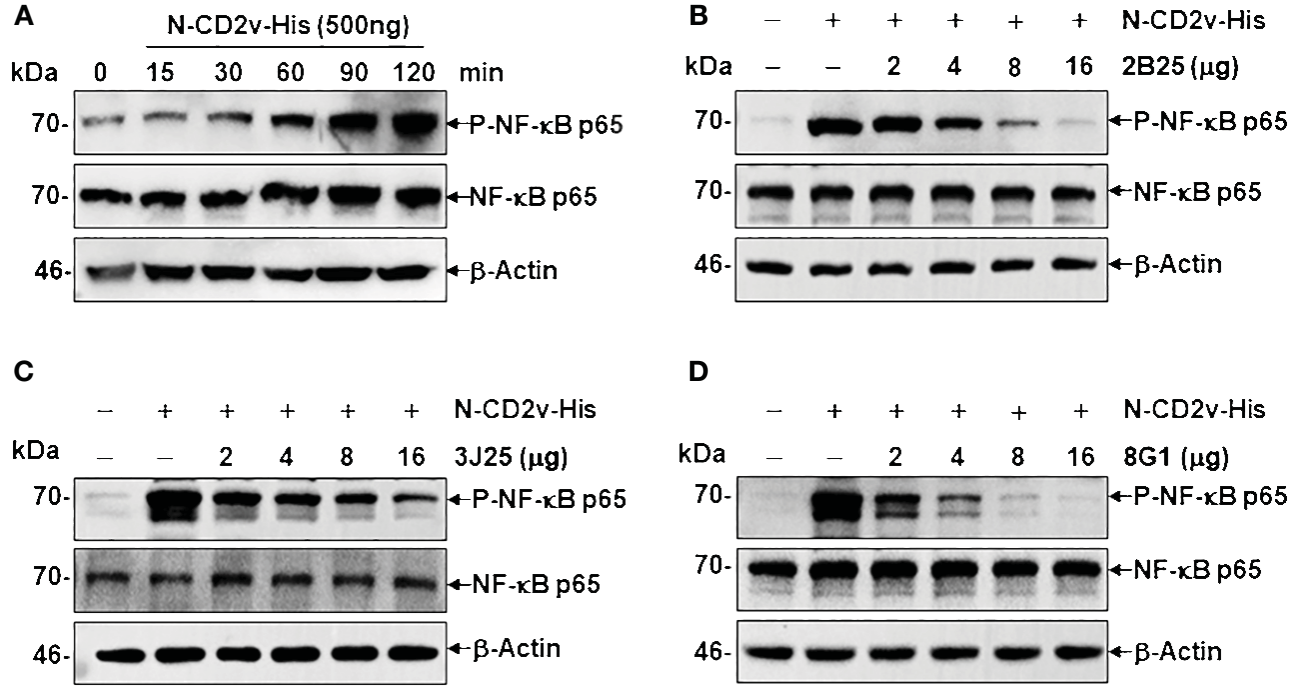
Anti-CD2v mAbs inhibit CD2v-induced NF-κB activation. **(A)** Time course experiment of CD2v-induced NF-κB activation. PK-15 cells were incubated with 500 ng of N-CD2v-His protein for various durations (0, 15, 30, 60, 90, and 120 min). Whole-cell lysates were separated by western blotting and probed with antibodies against p-NF-κB p65, NF-κB p65, and β-actin. **(B–D)** Effect of mAbs (2B25, 3J25, and 8G1) on CD2v-induced NF-κB activation. Purified N-CD2v-His (500 ng) was preincubated with each of the three mAbs (at doses of 2–16 µg) separately for 30 min at 37°C. The mixture was then co-cultured with PK-15 cells for 90 min at 37°C, and p-NF-κB p65, NF-κB p65, and β-actin were detected.

CD2v has been shown to induce activation and nuclear translocation of NF-κB p65 in swine peripheral blood mononuclear cells and macrophages (25). To assess the effects of the mAbs on the NF-κB signaling pathway, we conducted a time course experiment to determine the duration regarding CD2v-induced NF-κB activation. Treatment with purified N-CD2v-His protein resulted in the phosphorylation of NF-κB p65 (70 kDa), which exhibited a continuous increase from 15 to 120 min (**Figure 9A**). Based on these findings, we selected a 90-minute incubation period for PK-15 cells with purified N-CD2v-His protein, either alone or in combination with the anti-CD2v mAbs (2B25, 3J25, or 8G1). All three mAbs exhibited significantly and dose-dependently inhibition of CD2v-mediated NF-κB phosphorylation in PK-15 cells (**Figures 9B-D**). Furthermore, when used individually, neither the three mAbs nor the mIgG isotype control were capable of stimulating NF-κB activation (**Supplementary Figure S5**). These findings indicate that the anti-CD2v mAbs specifically interfered with the ability of soluble CD2v to induce NF-κB p65 activation in PK-15 cells.

## 4 Discussion

Associated with severe economic losses, ASF is a devastating and highly contagious hemorrhagic disease that threatens the swine industry worldwide. The identification of antigenic target proteins is key for mitigating the risks associated with ASFV (38). The outer envelope, which forms during ASFV budding from the host cytoplasm, plays a pivotal role in the virus’s pathogenicity (49). Deletion of the *EP402R* gene, which encodes outer envelope protein CD2v, in various ASFV strains reduces their pathogenicity (50–52). Furthermore, CD2v has been identified as a critical antigen in the immunoprotective response against ASFV, as indicated by numerous studies (22, 28–30). Immunization with recombinant baculovirus carrying the ASFV *EP402R* gene effectively protects pigs from subsequent challenges with virulent strains, suggesting the potential of the CD2v protein to activate cytotoxic T lymphocytes (53).

This study aimed to develop a highly specific and sensitive mAb targeting the ASFV outer envelope protein CD2v. CD2v, with a molecular weight of 105–110 kDa after glycosylation, presents a challenge due to its numerous glycosylation sites, making complete glycosylation using prokaryotic expression systems difficult (43, 54). As the immunogenicity of CD2v is pivotal for obtaining high-titer mAbs, we opted for the Expi293F eukaryotic expression system to express the recombinant N-CD2v-His protein (extracellular region of CD2v). This system offers superior post-translational modifications, particularly glycosylation, ensuring that the antigens expressed closely resemble the *in vivo* structure of CD2v. SDS-PAGE revealed that our recombinant protein had a molecular weight of approximately 70–100 kDa (**Figures 1B, C**), significantly larger than the predicted molecular weight of 23 kDa. This finding indicates significant glycosylation of the recombinant N-CD2v-His protein, consistent with previous findings (22, 23, 43, 55). It should be noted that higher molecular weight bands (120 kDa and >180 kDa) in the SDS-PAGE gel but not detect in the western blotting analysis. These bands could potentially be contaminants from the purification process. Another possibility is that they may arise from the oligomerization of CD2v, which could hinder accessibility to the His epitope. While there is currently no conclusive evidence for the oligomerization of the CD2v protein, we plan to conduct further experiments to confirm this in future research.

Next, the recombinant N-CD2v-His protein was utilized to immunize BALB/c mice. These mice had high-titer antibodies that specifically bound to CD2v, indicating the excellent immunogenicity of N-CD2v-His (**Figure 1D**). Subsequently, using hybridoma technology, we obtained four mAbs (2B25, 3J25, 7B1, and 8G1) with robust binding activity (**Figure 2A**). Importantly, all four mAbs exhibited specifically recognition of CD2v, while showing no binding to hCD2 or mCD2, despite the high similarity between hCD2/mCD2 and CD2v (18) (**Figure 2B**). Furthermore, it was observed that mAbs 2B25, 3J25, and 8G1 did not display significant binding to the ASFV structural proteins P30, P72, and pp62 (**Figure 2C**). These findings indicate that the screened mAbs, particularly 2B25, 3J25, and 8G1, are specifically targeted towards the CD2v antigen.

BLI showed that all four mAbs exhibited a high-affinity K_D_ to the CD2v antigen at the nanomolar level (**Figure 3**); 3J25 demonstrated the strongest binding affinity, while 7B1 exhibited the weakest. Notably, the order of binding strength according to BLI (3J25, 2B25, 8G1, 7B1) slightly differed from that according to indirect ELISA (2B25, 3J25, 8G1, 7B1). This variance may be attributable to the direct fixation of the antigen onto the ELISA plate (potentially changing the antigen conformation), while the antigen was immobilized onto the Ni-TNA biosensor in the BLI experiments (closely resembling the actual *in vivo* conditions). Following this, we selected the three mAbs with the highest affinity (3J25, 2B25, and 8G1) for further experiments.

Western blotting of the three mAbs indicated that they reacted with the denatured full-length CD2v protein (containing linear B-cell epitopes) (**Figure 4A**). Interestingly, all three mAbs were able to bind to glycosylated CD2v, while only 3J25 and 8G1 (not 2B25) recognized deglycosylated CD2v. This suggested that the binding of 2B25 depended on the glycosylation status of the CD2v surface. Furthermore, IFA demonstrated that all three mAbs recognized the full-length CD2v-GFP fusion protein and co-localized with green fluorescence (**Figure 4B**), confirming their ability to recognize naturally glycosylated epitopes of CD2v.

To develop a sandwich ELISA detection kit, we first assessed epitope overlap among the three mAbs using competitive ELISA. 2B25, 3J25, and 8G1 did not exhibit competition with each other in binding to CD2v (**Figures 5A-C**), indicating no epitope overlap. Based on these results, pairwise sandwich ELISAs were performed, which revealed that using 3J25 as the capture antibody and 2B25-biotin as the detection antibody led to the successful detection of CD2v at ng/mL levels. (**Figures 5D, E**).

Although multiple linear B-cell epitopes of CD2v have recently been identified (34–40), their functions remain unknown (**Table 1**). To analyze the antigenic epitopes recognized by our anti-CD2v mAbs, we designed and constructed five CD2v extracellular domain truncation mutants (D1–D5) (**Figures 6A-C**). Our results indicated that mAbs 2B25 and 3J25 primarily target the D1 region, while 8G1 primarily targets the D4 region. Moreover, the fine epitope recognition by 2B25 and 3J25 differed. To further differentiate the epitopes recognized by these three mAbs, we designed 11 overlapping polypeptides spanning the D1 and D4 regions (**Figure 7**). Through peptide-based ELISA, we identified two novel epitopes. The first epitope, recognized by mAb 3J25, was identified as ^25^TIILDSNITNDNN^37^. It overlapped with the linear epitope of amino acids 28–51 in the CD2v extracellular domain, which was identified by Zhang’s group (35). The other epitope, recognized by mAb 8G1, was identified as ^141^LNINDTFVKYTNE^153^. It contained the epitope ^147^FVKYT^151^, which was identified by Wang’s group (34), and partially overlapped with the epitope ^148^VKYTNESILE^157^, which was identified by the same group in a separate study (36). Interestingly, we could not identify the B-cell linear epitopes of mAb 2B25, which may recognize glycosylated epitopes in the D1 region. This is likely because directly synthesized peptides lack glycosylation modifications. Furthermore, multiple sequence alignment revealed that the two identified epitopes (^25^TIILDSNITNDNN^37^ and ^141^LNINDTFVKYTNE^153^) are conserved in ASFV genotype II strains, particularly in Chinese strains. This indicates that the two epitopes are an important feature of this branch of ASFV and could be used for the differential diagnosis of different strains (**Figure 8**). These findings may have implications for novel ASFV vaccine design.

During *in vivo* infection, pigs infected with highly virulent strains of ASFV exhibit elevated systemic production of IFN, TNF-α, IL-1α, IL-1β, and IL-6, mainly facilitated by NF-κB or alternative transcription factors (56–58). Notably, recent research has shown that ASFV CD2v induces NF-κB-dependent IFN-β and ISGs transcription in swine PK15 cells (25). when purified N-CD2v-His was preincubated with an anti-CD2v mAb (2B25, 3J25, or 8G1) before incubation for 90 min with PK-15 cells (**Figures 9B-D**), the mAbs significantly and dose-dependently inhibited CD2v-dependent NF-κB activation. This implies that anti-CD2v antibodies might be an important immune mechanism for neutralizing CD2v. These findings underscore the potential significance of targeting CD2v in the development of immunotherapeutic strategies against ASFV infection.

In summary, we successfully expressed recombinant CD2v protein in eukaryotic cells and generated mAbs (**Table 2**) that recognize two novel linear CD2v epitopes and one glycosylated epitope. By utilizing the mAbs 2B25 and 3G25, we established a highly sensitive sandwich ELISA to detect the CD2v antigen. These mAbs and their target epitopes are likely to be valuable for studying the structure and function of CD2v, differentiating between virus strains, and other applications. Additionally, the identified B cell epitopes may serve as candidate vaccine antigens for preventing infections by prevalent ASFV strains in China. Nonetheless, our study does have certain limitations that need to be addressed in future research. Specifically, further refinement of the epitope information, determination of the specific amino acids within the glycosylated epitope of CD2v, and additional evaluation of the specificity and sensitivity of the sandwich ELISA (using ASFV reference strains and infected pig samples) are required. Additionally, the identified mAbs need further validation to confirm their specificity in blocking ASFV infection in relevant animal models or *in vitro* systems. These areas will be the primary focus of our future research endeavors.

## Data availability statement

The original contributions presented in the study are included in the article/**Supplementary Material**. Further inquiries can be directed to the corresponding authors.

## Ethics statement

The animal study was approved by Ethics Committee of the Academy of Beijing Institute of Pharmacology and Toxicology. The study was conducted in accordance with the local legislation and institutional requirements.

## Author contributions

RF: Data curation, Formal Analysis, Investigation, Methodology, Writing – original draft. ZW: Data curation, Investigation, Methodology, Writing – review & editing. MZ: Data curation, Investigation, Methodology, Software, Writing – review & editing, Validation. SJ: Data curation, Investigation, Methodology, Writing – review & editing. ZJ: Data curation, Investigation, Methodology, Writing – review & editing. YW: Investigation, Methodology, Writing – review & editing. JC: Data curation, Investigation, Methodology, Writing – review & editing. GC: Investigation, Writing – review & editing, Formal Analysis, Methodology. HX: Investigation, Writing – review & editing, Data curation, Methodology. YXW: Funding acquisition, Investigation, Writing – review & editing, Formal Analysis, Methodology. YS: Investigation, Writing – review & editing. JF: Investigation, Supervision, Writing – review & editing, Data curation, Software. BS: Supervision, Writing – review & editing, Conceptualization, Investigation, Methodology. YZ: Investigation, Supervision, Writing – review & editing, Conceptualization, Methodology. YH: Investigation, Supervision, Writing – review & editing, Conceptualization, Methodology. JW: Conceptualization, Funding acquisition, Methodology, Project administration, Supervision, Visualization, Writing – original draft, Writing – review & editing, Investigation.
