# Supplementary material for: Development of novel monoclonal antibodies for blocking NF-κB activation induced by CD2v protein in African swine fever virus
Source: Front Immunol. 2024 May 23;15:1352404. doi: 10.3389/fimmu.2024.1352404 (PMC11153791; doi:10.3389/fimmu.2024.1352404)
Supplement: Supplementary file 1 [file Image_1.pdf]

Supplementary Material

Supplementary Figures

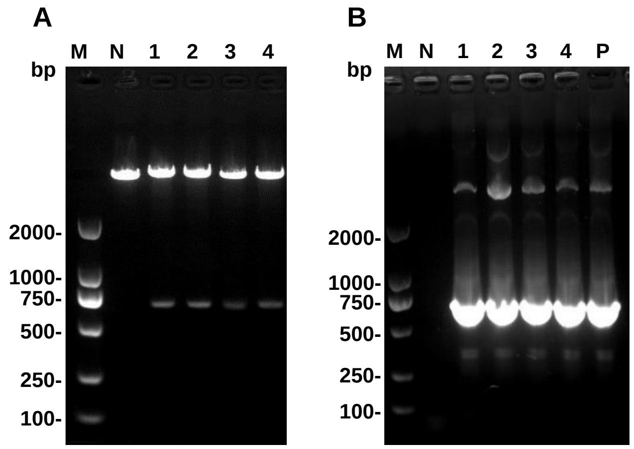

Figure S1 Construction of eukaryotic expression plasmid expressing the CD2v extracellular domain. (A) pcDNA3.1-N-CD2v-His plasmid digestion with *Not*I/*Xba*I restriction enzymes. (B) Colony PCR identification. N: negative control (empty plasmid); M: DNA molecular markers (DL-2000); 1–4: 4 randomly selected clones; P: positive control.
