# Supplementary figures and images for: Development of novel monoclonal antibodies for blocking NF-κB activation induced by CD2v protein in African swine fever virus
Source: Front Immunol. 2024 May 23;15:1352404. doi: 10.3389/fimmu.2024.1352404 (PMC11153791; doi:10.3389/fimmu.2024.1352404)

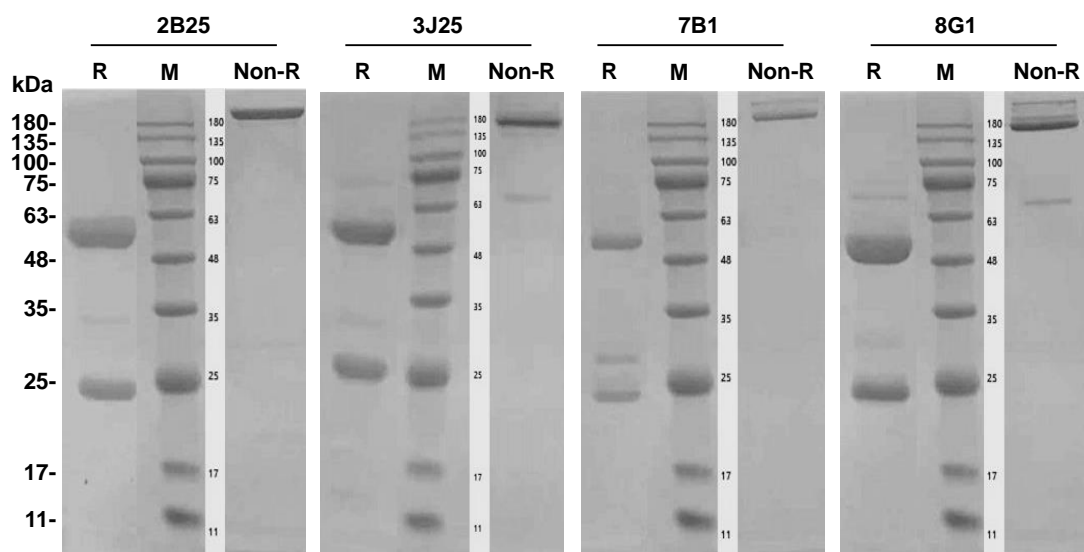

**Figure S2** Identification of four purified mAbs by SDS-PAGE. R. reducing; M. marker; Non-R. non-reducing.

Supplement: Supplementary file 2 [file Image_2.pdf]
