# Supplementary material for: Development of novel monoclonal antibodies for blocking NF-κB activation induced by CD2v protein in African swine fever virus
Source: Front Immunol. 2024 May 23;15:1352404. doi: 10.3389/fimmu.2024.1352404 (PMC11153791; doi:10.3389/fimmu.2024.1352404)
Supplement: Supplementary file 3 [file Image_3.pdf]

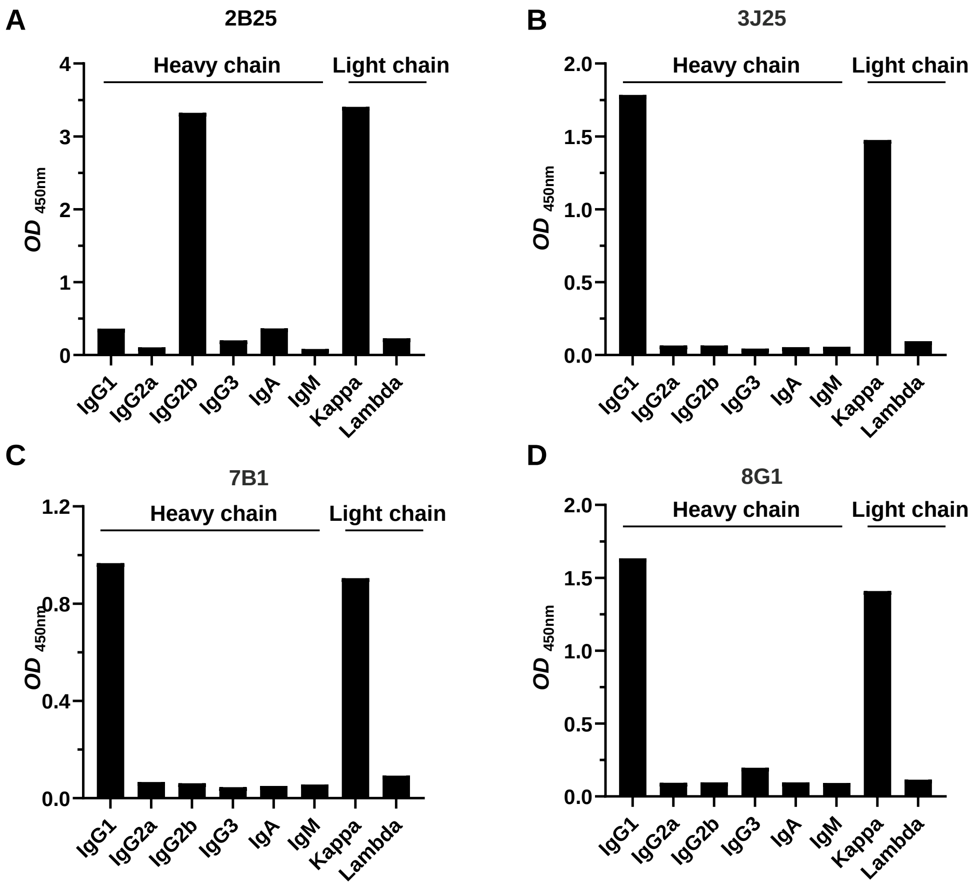

Figure S3 Identification of antibody subtypes against CD2v. (A–D) Heavy- and light-chain isotype identification for mAbs 2B25, 3J25, 7B1, and 8G1, respectively.
