# Supplementary material for: Development of novel monoclonal antibodies for blocking NF-κB activation induced by CD2v protein in African swine fever virus
Source: Front Immunol. 2024 May 23;15:1352404. doi: 10.3389/fimmu.2024.1352404 (PMC11153791; doi:10.3389/fimmu.2024.1352404)
Supplement: Supplementary file 4 [file Image_4.pdf]

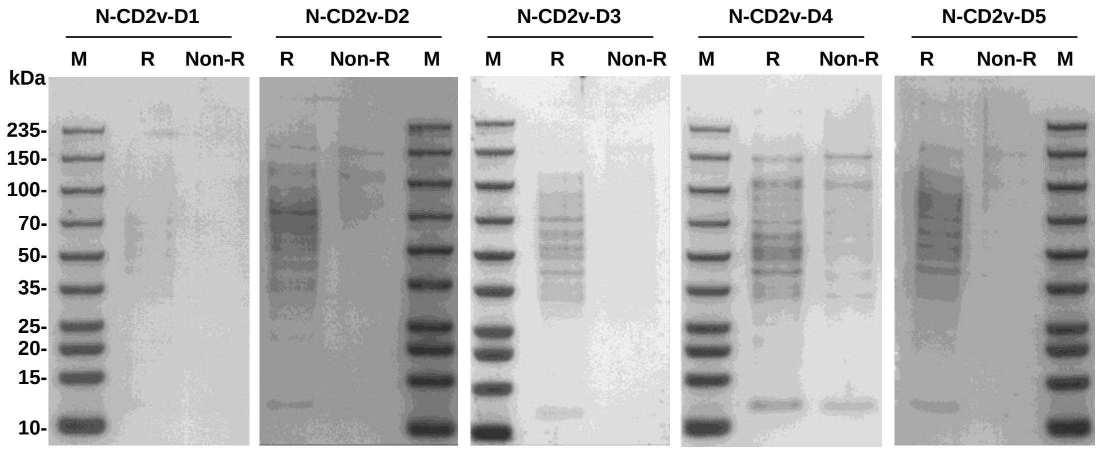

Figure S4 SDS-PAGE analysis of five CD2v extracellular domain truncation mutants (N-CD2v-D1–D5) in 4%-12% gradient gels. R: reducing; M. protein marker; Non-R: non-reducing.
