# Supplementary material for: Development of novel monoclonal antibodies for blocking NF-κB activation induced by CD2v protein in African swine fever virus
Source: Front Immunol. 2024 May 23;15:1352404. doi: 10.3389/fimmu.2024.1352404 (PMC11153791; doi:10.3389/fimmu.2024.1352404)
Supplement: Supplementary file 5 [file Image_5.pdf]

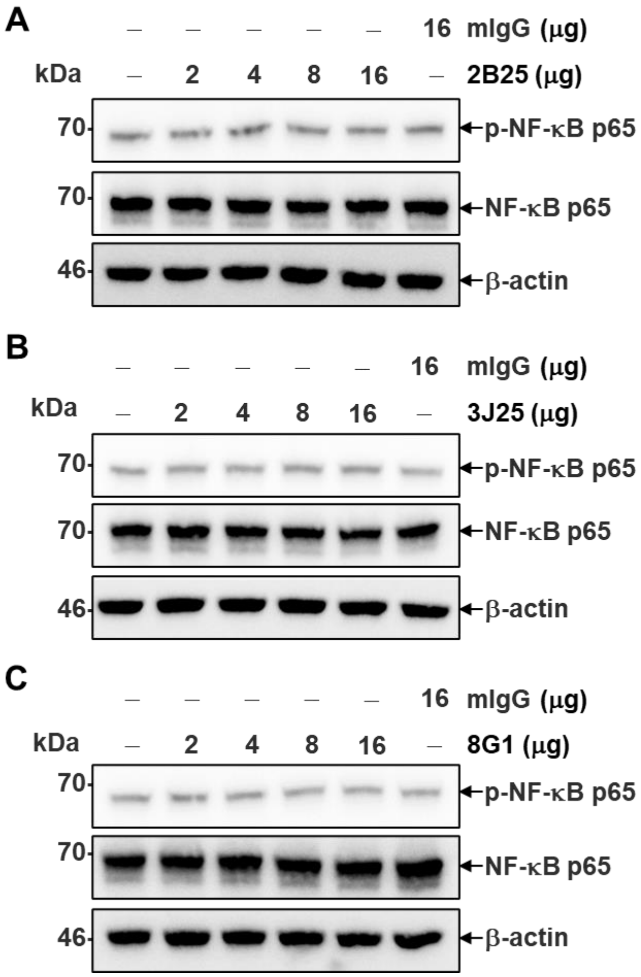

Figure S5 Impact of Anti-CD2v mAbs and mouse IgG (mIgG) on NF-κB Activation. PK-15 cells were treated with mAbs at doses ranging from 2 to 16 µg, as well as mIgG at 16 µg, for 90 minutes at 37°C, respectively. Following incubation, western blotting was performed to assess the expression levels of p-NF-κB p65, NF-κB p65, and β-actin, as depicted in panels (A) 2B25, (B) 3J25, and (C) 8G1.
