# Supplementary material for: Development of novel monoclonal antibodies for blocking NF-κB activation induced by CD2v protein in African swine fever virus
Source: Front Immunol. 2024 May 23;15:1352404. doi: 10.3389/fimmu.2024.1352404 (PMC11153791; doi:10.3389/fimmu.2024.1352404)
Supplement: Supplementary file 6 [file Table_1.pdf]

Supplementary Tables

Table S1 List of 42 ASFV epidemic strains used for sequence conservation analysis.

No.	Isolate strains	Country	Year	Protein id.	Accession no.	Genotype
01	Benin 97/1	Benin	1997	YP_009703511.1	NC_044956.1	I
02	Nu1979	Italy	1979	UCX48528.1	MW723481.1	I
03	25185_2008	Italy	2008	UCX58457.1	MW788410.1	I
04	30322	Italy	2013	UCX53830.1	MW736600.1	I
05	BA71V	Spain	1971	NP_042752.1	NC_001659.2	I
06	E75	Spain	1975	YP_009703827.1	NC_044958.1	I
07	NHV	Portugal	1968	YP_009702625.1	NC_044943.1	I
08	OURT 88/3	Portugal	1988	YP_009703666.1	NC_044957.1	I
09	K-49	Zaire	1949	AJB28366.1	KM609339.1	I
10	ASFV Belgium 2018/1	Belgium	2018	VFV47992.1	LR536725.1	II
11	Belgium/Etalle/wb/2018	Belgium	2018	QED90501.1	MK543947.1	II
12	Pig/HLJ/2018	China	2018	QBH90546.1	MK333180.1	II
13	China/2018/AnhuiXCGQ	China	2018	AYW34030.1	MK128995.1	II
14	DB/LN/2018	China	2018	QBH90731.1	MK333181.1	II
15	ASFV-wbBS01	China	2018	QDL88089.1	MK645909.1	II
16	CN/2019/InnerMongolia-AES01	China	2019	QIA61472.1	MK940252.1	II
17	GZ201801	China	2018	QLF78587.1	MT496893.1	II
18	ASFV Wuhan 2019-1	China	2019	QIE06850.1	MN393476.1	II
19	CADC_HN09	China	2019	UFD97826.1	MZ614662.1	II
20	China/GX/201909	China	2019	UTS69469.1	OM986272.1	II
21	China/GX/201915	China	2019	UTS69473.1	OM986278.1	II
22	China/GX/201937	China	2019	UTS69493.1	OM986300.1	II
23	China/GX/201947	China	2019	UTS69501.1	OM986310.1	II
24	China/GX/202011	China	2020	UTS69514.1	OM986324.1	II
25	China/GX/202013	China	2020	UTS69516.1	OM986326.1	II
26	SY-1	China	2022	UYC33132.1	OM161110.1	II
27	Estonia 2014	Estonia	2014	SPS73481.1	LS478113.1	II
28	ASFV Georgia 2007/1	Georgia	2007	CAD2068420.1	FR682468.2	II
29	Georgia 2008/1	Georgia	2018	AZP54004.1	MH910495.1	II
30	ASFV_HU_2018	Hungary	2018	QGV56849.1	MN715134.1	II
31	ASFV/LT14/1490	Lithuania	2014	QEY87864.1	MK628478.1	II
32	MAL/19/Karong	Malawi	2019	QXP50017.1	MW856068.1	II

33	Pol17_55892_C754	Poland	2017	QOW02555.1	MT847620.1	II
34	ASFV/Amur 19/WB-6905	Russia	2019	QUQ60163.1	MW306190.1	II
35	ASFV_NgheAn_2019	Vietnam	2019	QOY24367.1	MT180393.1	II
36	Tanzania/Rukwa/2017/1	Tanzania	2017	CAD0059509.1	LR813622.1	II
37	ASFV/Kyiv/2016/131	Ukraine	2017	QED21622.1	MN194591.1	II
38	SPEC_57	South Africa	1985	QGM12912.2	MN394630.3	VIII
39	Ken06.Bus	Kenya	2006	YP_009702957.1	NC_044946.1	IX
40	R35	Uganda	2018	AXB49977.1	MH025920.1	IX
41	Ken05/Tk1	Kenya	2005	KM111294.1	AJL34072.1	X
42	TSP80	Tanzania	1980	AJB28401.1	KM609359.1	X
